# Deficient Autophagy in Microglia Aggravates Repeated Social Defeat Stress-Induced Social Avoidance

**DOI:** 10.1155/2022/7503553

**Published:** 2022-02-16

**Authors:** Mai Sakai, Zhiqian Yu, Ryo Hirayama, Masa Nakasato, Yoshie Kikuchi, Chiaki Ono, Hiroshi Komatsu, Miharu Nakanishi, Hatsumi Yoshii, David Stellwagen, Tomoyuki Furuyashiki, Masaaki Komatsu, Hiroaki Tomita

**Affiliations:** ^1^Department of Psychiatry, Graduate School of Medicine, Tohoku University, Sendai, Japan; ^2^Department of Psychiatric Nursing, Graduate School of Medicine, Tohoku University, Sendai, Japan; ^3^Department of Neurology and Neurosurgery, Centre for Research in Neuroscience, The Research Institute of the McGill University Health Center, Montreal, Canada H3G 1A4; ^4^Division of Pharmacology, Kobe University Graduate School of Medicine, Kobe, Japan; ^5^Department of Physiology, Juntendo University Graduate School of Medicine, Tokyo, Japan; ^6^Department of Disaster Psychiatry, Graduate School of Medicine, Tohoku University, Sendai, Japan

## Abstract

Major depressive disorder (MDD) is associated with repeated exposure to environmental stress. Autophagy is activated under various stress conditions that are associated with several diseases in the brain. This study was aimed at elucidating the autophagy signaling changes in the prefrontal cortex (PFC) under repeated social defeat (RSD) to investigate the involvement of microglial autophagy in RSD-induced behavioral changes. We found that RSD stress, an animal model of MDD, significantly induced initial autophagic signals followed by increased transcription of autophagy-related genes (Atg6, Atg7, and Atg12) in the PFC. Similarly, significantly increased transcripts of ATGs (Atg6, Atg7, Atg12, and Atg5) were confirmed in the postmortem PFC of patients with MDD. The protein levels of the prefrontal cortical LC3B were significantly increased, whereas p62 was significantly decreased in the resilient but not in susceptible mice and patients with MDD. This indicates that enhanced autophagic flux may alleviate stress-induced depression. Furthermore, we identified that FKBP5, an early-stage autophagy regulator, was significantly increased in the PFC of resilient mice at the transcript and protein levels. In addition, the resilient mice exhibited enhanced autophagic flux in the prefrontal cortical microglia, and the autophagic deficiency in microglia aggravated RSD-induced social avoidance, indicating that microglial autophagy involves stress-induced behavioral changes.

## 1. Introduction

Major depressive disorder (MDD) is an important social issue that can potentially lead to suicide, and lifetime prevalence estimates are usually high in the general population [1, 2]. Repeated environmental stress is w5idely accepted to be involved in the pathogenesis of MDD, promoting its onset or recurrence [3]. Social activity is also avoided by patients with MDD. Social contacts provoke anxiety and depression, making minor stressors overwhelming [4]. Animals exposed to repeated stress have been used to understand the pathophysiology of MDD. For instance, repeated social defeat (RSD) stress causes a robust depression-like phenotype marked by anhedonia, anxiety, and social avoidance behaviors, and these behaviors are helpful for elucidating individual differences [5]. In the central nervous system (CNS), the prefrontal cortex (PFC) mediates the emotional influences on cognitive processes [6]. The PFC circuits are involved in stress responses in mice and patients [7, 8].

Recent studies have suggested an association between MDD and autophagy. Autophagy signaling carries its components into the intracellular digestive system and lysosomes and degrades them to promote survival [9]. Previous studies showed increased expression of autophagy-related genes in mononuclear cells in patients with MDD [10]. Attenuation of the mechanistic target of rapamycin (mTOR) signaling in the postmortem brains of depressed patients has been reported [11]. In animal models, chronic mild unpredictable stress in mice has been reported to enhance hippocampal autophagy [12]. Moreover, inhibition of autophagy plays a protective role in reducing depressive-like behavior in rats [13]. Astrocytic autophagic flux involves mitochondrial clearance in a chronic mild stress murine model of depression [14]. These findings suggest that abnormalities in autophagy and subsequent functional changes in the brain are involved in stress-induced depressive behavior.

Although several studies have focused on changes in brain structure and function, we focused on the role of microglia in the current study. Microglia are major immune cells in the central nervous system (CNS) [15]. Their activation has been involved in various psychiatric disorders, including MDD [16]. The involvement of microglia in stress is evident, including changes in microglial density in patients with MDD patients [17] and microglial activation in suicidal and affective disorder patients [18, 19]. Animal studies have revealed altered microglial morphology and higher resilience to stress-induced depression-like behavior in microglia-deficient mice [20]. RSD-induced avoidance is caused by microglial activation through toll-like receptors [21]. The microglial inflammatory response has led to an understanding of this pathology. However, it remains poorly understood whether microglial autophagy is also associated with immune response and behavioral changes related to stress and MDD. For instance, microglial autophagy inhibits microglia-derived TNF-*α* and enhances M1 but reduces M2 markers [22]. Deficient microglial autophagy impairs synaptic pruning and causes autism spectrum disorder-like behavior [23]. Microglial Atg5-deficient mice under chronic unpredictable stress during pregnancy showed decreased behavioral response to the antidepressant fluoxetine at one month postpartum [24]. However, the role of autophagy in microglia-driven behavioral changes in response to chronic stress has not yet been examined.

The current study was aimed at elucidating the autophagy signaling changes in the PFC under RSD to investigate the involvement of microglial autophagy in RSD-induced behavioral changes. We analyzed the transcripts of the autophagy-related gene (Atg) and protein levels of autophagosome markers in mouse PFC microglia under RSD stress. We also investigated the transcripts of ATGs in the postmortem PFC of the microarray database of patients with MDD. Furthermore, the microglial Atg7-knockout mice were used to evaluate the involvement of microglial autophagy in RSD-induced behavioral changes.

## 2. Materials and Methods

All experimental protocols were performed in accordance with the Guidelines for the Care of Laboratory Animals of Tohoku University Graduate School of Medicine (Sendai, Japan).

### 2.1. Animals

For all experiments, 8- to 12-week-old male mice were used. C57BL/6J and Slc:ICR (CD-1) mice were purchased from SLC Japan Inc. (Shizuoka, Japan). The mice were individually housed and maintained on a 12 : 12 h light/dark schedule with ad libitum access to food and water throughout the experimental period. The animals were acclimated for one week in our animal facility. Microglial-specific GFP-expressing CX3CR1^GFP/+^ [25] and microglial ATG7-deficient CX3CR1-Cre+;Atg7^flox/flox^ (Cre^+^;Atg^flox/flox^) mice were used in the current experiments. CX3CR1^GFP/GFP^ was obtained from the Jackson Laboratory and crossed with C57BL/6J mice. Floxed ATG7 mice obtained from RIKEN (RBRC02759) are generated by Komatsu et al. [26] and crossed with Tg(Cx3cr1-Cre)MW126Gsat mice [27] generated by Heintz (the Rockefeller University, GENSAT); Cx3cr1-Cre mouse [28] lines were generated at Tohoku University for more than ten generations. After weaning on postnatal days (PNDs) 21–28, all mice were housed socially in same-sex groups in a temperature-controlled environment under a 12 : 12 h light/dark cycle (lights on at 09:00 h) with ad libitum access to water and food. Genomic DNA extracted from mouse tails was used for the standard PCR genotyping.

### 2.2. Repeat Social Defeat Stress (RSD)

The RSD procedure was performed as previously reported [5]. In brief, clear rectangular cages (26.7 × 48.3 cm × 15.2 cm) with a clear perforated Plexiglas divider (0.6 × 45.7 × 15.2 cm) (cat. no. PC10196HT) and paired steel wire tops (cat. no. WBL1019 MMB) were purchased from Allentown Inc. (PA, USA). Social interaction open-field test boxes with opaque Plexiglas (42 × 42 × 42 cm) were custom-ordered (Latest Science Corp., Sendai, Japan). Mice were exposed to a different CD1 aggressor mouse for 10 min daily for 10 days by removing the clear perforated Plexiglas divider. After the last exposure session, all the mice were housed individually.

### 2.3. Social Interaction Test (SIT)

On day 11, the SIT [5] was performed to identify subgroups of mice that were susceptible or resilient to social defeat stress. This was accomplished by placing the mice in an open-field test box containing an empty wire mesh cage (10 × 4.5 cm) located at one end. The social interaction of the mice was measured for 2.5 min, followed by 2.5 min in the presence of an unfamiliar aggressor confined in the wire-mesh cage. The “interaction zone” of the test arena encompassed a 14 × 24 cm rectangular area projecting 8 cm around the wire-mesh enclosure. The duration spent by the subjects in the “interaction zone” was recorded using a video camera. The interaction ratio was calculated as the time spent in the interaction zone with an aggressor or time spent in the interaction zone without an aggressor. An interaction ratio of 1 was set as the cutoff, whereby mice with scores < 1 were defined as “susceptible mice” to social defeat stress and those with scores ≥ 1 were defined as “resilient mice.”

### 2.4. Elevated Plus Maze Test (EPM)

The apparatus consisted of a plus-shaped maze with two opposing open arms (25 × 5 cm) and two opposing closed arms (25 × 5 cm, surrounded by 17 cm high walls) that extended from a central platform (5 × 5 cm) to form a cross shape. The maze was elevated 40 cm above the floor. The mice were individually placed in the center platform facing an open arm and allowed to freely explore the apparatus for 10 min. The time spent in the open arms and the number of open and closed arm entries were automatically measured using ANY-maze video tracking software (Stoelting Co., Wood Dale, IL). The number of open and closed arm entries was combined to yield a measure of total entries, which reflected the general exploratory activity during the test.

### 2.5. Sucrose Preference Test (SPT)

The SPT employed a two-bottle, free-choice sucrose consumption paradigm using previously described methods [29]. The mice were habituated to drink water from two tubes with stoppers fitted with ball-point sippers (Ancare, Bellmore, NY, USA) for two days. They were then exposed to 1% sucrose or drinking water following habituation for three consecutive days. The weights of the water- or sucrose-containing bottles were measured before and at the end of this period. Sucrose preference was determined using the following equation:

(1) $$\begin{array}{c} \text{Sucrose preference}=\frac{\text{sucrose day }1-\text{sucrose day }2\;}{\left(\text{sucrose day }1-\text{sucrose day }2\right)+\left(\text{water day }1-\text{water day }2\right)}\times 100. \end{array}$$

### 2.6. Quantitative Real-Time PCR

Total RNA was extracted from PFC and used as a template for cDNA synthesis using random primers and the SuperScript VILO cDNA synthesis kit (Invitrogen, Carlsbad, CA, USA). The relative copy number of each transcript in each cDNA sample was measured using specific primers and iQ SYBR Green Supermix (Bio-Rad Inc., Hercules, CA, USA). A standard curve was constructed for each assay to adjust for differences in the amplification efficiency of the primer sets. 18S rRNA was used as an internal control for normalization. The forward and reverse primers for 18S were 5′-GTAACCCGTTGAACCCCATT-3′ and 5′-CCATCCA ATCGGTAGTAGCG-3′, respectively. The forward and reverse primers for Atg5 were 5′-GGAGAGAAGAGGAGCCAGGT-3′ and 5′-TGTTGCCTCCACTGAACTTG-3′, respectively. The forward and reverse primers for Beclin1 (Atg6) were 5′-GGCCAATAAGATGGGTCTGA-3′ and 5′-GCTGCACACAGTCCAGAAAA-3′, respectively. The forward and reverse primers for Atg7 were 5′-TCCGTTGAAGTCCTCTGCTT-3′ and 5′-CCACTGAGGTTCACCATCCT-3′, respectively. The forward and reverse primers for Atg12 were 5′-TCCGTTGAAGTCCTCTGCTT-3′ and 5′-CAGCACCGAAATGTCTCTGA-3′, respectively. The forward and reverse primers for Lc3a were 5′-CATGAGCGAGTTGGTCAAGA-3′ and 5′-TTGACTCAGAAGCCGAAGGT-3′, respectively. The forward and reverse primers for Lc3b were 5′-CCCACCAAGATCCCAGTGAT-3′ and 5′-CCAGGAACTTGGTCTTGTCCA-3′, respectively. The forward and reverse primers for Fkbp5 were 5′-GAGTCTGCGAAAGGACTTGG-3′ and 5′-GTGGGTTCTACATCGGCACT-3′, respectively.

### 2.7. Microarray Analyses of Postmortem Human Brains

The microarray data of postmortem brain tissues (Brodmann area 10: anterior prefrontal cortex) from patients with schizophrenia and healthy controls (SOFT files and CEL files) were downloaded from the Gene Expression Omnibus (GEO) repository (GSE92538) housed at the National Center for Biotechnology Information (NCBI) on their FTP site (ftp://ftp.ncbi.nih.gov/pub/geo/). We used data from the postmortem dorsolateral PFC of patients with MDD. The SOFT and CEL files from the dataset GPL10526 (healthy subjects, *n* = 56; MDD patients, *n* = 29) [30], which included 54,120 probe sets, were imported into the BRB-Array Tools v4.6.0 Beta 1 software (https://brb.nci.nih.gov/BRB-ArrayTools/) [31]. Additionally, the dataset GPL17027 (healthy subjects, *n* = 111; MDD patients, *n* = 43) with only 12,334 probes were excluded. Signal intensities less than 50 and *P* values less than 0.05 were rejected. After normalizing the interarray variation among the 85 microarrays using quantile normalization, the significantly differentially expressed genes in each pairwise comparison were identified by a random variance *t*-test with the Benjamini–Hochberg false discovery (FDR) correction [31].

### 2.8. Immunohistochemical Analysis of Mouse Microglial Autophagy

Immunohistochemistry was performed using a standard method [15]. To determine the expression of markers of mouse microglia autophagy in vivo, the mice were divided into control, susceptible, and resilient groups after chronic stress exposure according to the methods described. In brief, mice were anesthetized with an intraperitoneal (i.p.) injection of pentobarbital (mg/kg, NEMBUTAL Injection Dainippon Pharmaceutical Co., Ltd., Japan) at 0.5 mg/kg and transcardially perfused with phosphate-buffered saline (FUJIFILM Wako Pure Chemical Corp. Osaka, Japan), followed by 4% paraformaldehyde phosphate buffer solution (FUJIFILM Wako Chemical Corp.). The brains were immersed in 4% paraformaldehyde for 24 h and changed to 30% sucrose for 24 h. After the brains were rapidly frozen in OCT compound (Sakura Finetek, Torrance, CA, USA), coronal brain sections of 30 *μ*m thickness were made using a cryostat (Carl Zeiss MicroImaging GmbH, Jena, Germany). PFC slices (30 *μ*m thick) dissected from frozen brains were reacted with the following antibodies: Alexa Fluor 647-conjugated rabbit anti-mouse LC3B antibody (1 : 250; Abcam, Cambridge, MA, USA), mouse anti-mouse p62/SQSTM1 (1 : 300; R&D Systems, Minneapolis, MN, USA), and mouse anti-mouse anti-FKBP51 antibody (Hi51B) (1 : 300; Abcam). The secondary antibody used was Alexa Fluor 594-conjugated anti-mouse IgG (1 : 300; Invitrogen). Nuclei present in the slices were stained with 4,6-diamidino-2-phenylindole (DAPI; Invitrogen). Images of cells were acquired using a fluorescence microscope (Axio Scope.A1; Carl Zeiss, Oberkochen, Germany). The levels of Cx3cr1, LC3B, and p62 signals were obtained using ImageJ 1.53K software (NIH Image, Bethesda, MD, USA).

### 2.9. Statistical Analysis

A two-tailed unpaired Student's *t*-test was used to evaluate the differences in the mean values between the two groups. One- or two-way analysis of variance (ANOVA) followed by Tukey's or Sidak post hoc tests was used for comparisons among more than two groups. Statistical analyses were performed using IBM SPSS Statistics for Windows, version 22.0 (IBM Japan, Tokyo, Japan).

## 3. Results

### 3.1. Repeated Social Defeat Stress-Induced Depressive-Like Behavior

**Figure 1:**
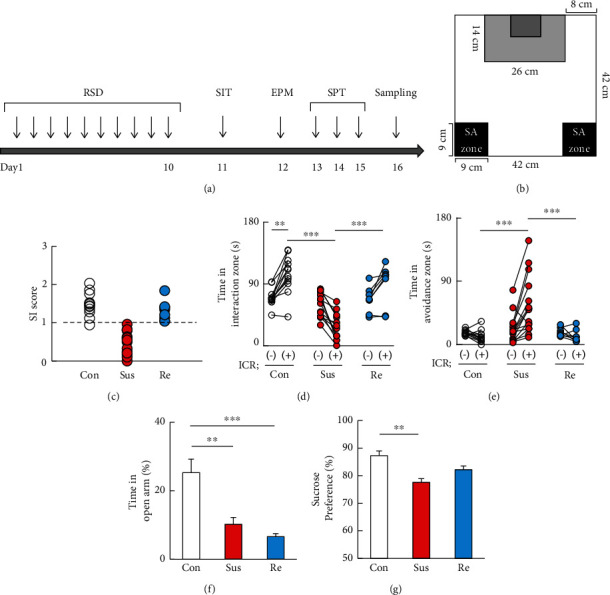
The effects of chronic restraint stress on behavior. (a) Schedule of behavioral experiments. Beginning at day 0 and concluding on day 10, each animal was assigned to the RSD group. From day 11 to day 15, the mice were subjected to SIT, EPM, and SPT each day, and brain samples were collected at day 16. (b) Definitions of the social interaction zone and the social avoidance zone (grey and black rectangles, respectively). (c) Horizontal scatterplot depicting the distribution of interaction ratios for control. The durations in the social interaction zone (d) and social avoidance zone (e) in wild-type (WT) mice with or without RSD. (f) The duration of the open arms in the elevated plus maze test as an index for anxiety of wild-type mice. (g) The proportion of the sucrose intake as an index for depression of wild-type mice after RSD. RSD: repeated social defeat; SIT: social interaction test; Con: control mice; Sus: susceptible mice; Re: resilient mice; SI: social interaction; SA: social avoidance. One-way ANOVA followed by Turkey's post hoc test was applied to all comparisons (Con, *n* = 12; Sus, *n* = 16; Res, *n* = 5). Data are presented as the mean ± SEM.

After ten days of RSD (Figure 1(a)), SIT with or without CD1 mouse exposure on day 11 (Figure 1(b)) separated the stressed mice into two groups: susceptible (*n* = 16) and resilient mice (*n* = 5) (Figure 1(c)). Avoidance behavior was analyzed by one-way ANOVA with Tukey's multiple comparison analyses, including nonstressed mice (controls; *n* = 12). SIT showed that nonstressed controls exhibited significantly increased time in the interaction zone when exposed to CD1 mice (white circle; *P* < 0.01) (*F*_5,60_ = 23.11, *P* < 0.0001; Figure 1(d)). Conversely, RSD-stressed susceptible mice (red circle; *P* < 0.01) showed significantly decreased time in the interaction zone (Figure 1(d)) but increased time in the avoidance zone (*F*_5,60_ = 7.939, *P* < 0.0001; Figure 1(e)) when exposed to CD1 mice compared with nonstressed controls (white circle; *P* < 0.001) and resilient mice (blue circle; *P* < 0.01). Anxiety and depressive-like behaviors were further evaluated by EPM and SPT, respectively, with nonstressed controls on four consecutive days (Figure 1(a)). One-way ANOVA followed by Tukey's multiple comparison analyses showed that our results were consistent with those of previous studies [29] in that both susceptible (*P* < 0.001) and resilient (*P* < 0.001) mice showed significantly less time spent in the open arm of the EPM compared with controls (*F*_2,30_ = 14.66, *P* < 0.001; Figure 1(f)). Furthermore, sucrose preference was significantly decreased in susceptible mice compared with controls (*P* < 0.01) (*F*_2,30_ = 18.00, *P* < 0.001; Figure 1(g)).

### 3.2. Increased Autophagy Signaling in the Prefrontal Cortex of Stressed Mice

**Figure 2:**
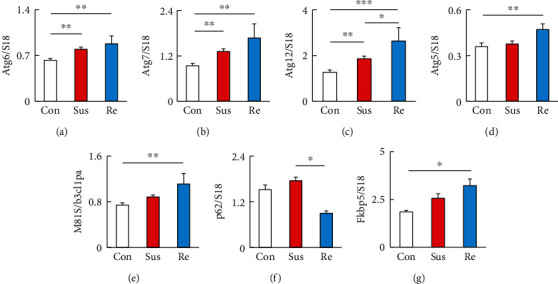
Repeated social defeat stress induced the expression of autophagic signaling in the prefrontal cortex. (a) Levels of the mRNA encoding the autophagic signaling marker Atg6 relative to those of S18. (b) Levels of the mRNA encoding the autophagic signaling marker Atg7 relative to those of S18. (c) Levels of the mRNA encoding the autophagic signaling marker Atg12 relative to those of S18. (d) Levels of the mRNA encoding the autophagic signaling marker Atg5 relative to those of S18. (e) Levels of the mRNA encoding the autophagic signaling marker Map1lc3b relative to those of S18. (f) Levels of the mRNA encoding the autophagic signaling marker p62 relative to those of S18. (g) Levels of the mRNA encoding the autophagic activator Fkbp5 relative to those of S18. Con: control mice; Sus: susceptible mice; Re: resilient mice. One-way ANOVA followed by Turkey's post hoc test was applied to all comparisons (Con, *n* = 12; Sus, *n* = 16; Res, *n* = 5). Data are presented as the mean ± SEM. ^∗^*P* < 0.05, ^∗∗^*P* < 0.01, and ^∗∗∗^*P* < 0.001 vs. Con or Re.

We examined the transcripts of major autophagy-related genes (Atg) in the PFC using real-time qRT-PCR. Stressed mice were divided into susceptible and resilient mice based on the results of SIT (control mice, *n* = 12; susceptible mice, *n* = 16; and resilient mice, *n* = 5). Beclin1/Atg6, an essential autophagy-promoting protein, is important for the localization of autophagic proteins to a preautophagosomal structure [32]. After one-way ANOVA with Tukey's post hoc analyses, *Atg6* transcripts in the PFC were significantly increased in both susceptible (*P* < 0.01) and resilient (*P* < 0.01) mice compared with controls (*F*_2,47_ = 7.317, *P* = 0.0017; Figure 2(a)). Atg7 is highly critical for autophagosome formation, which mediates ATG12-ATG5 complex formation, and the latter complex along with LC3-II [33]. *Atg7* transcripts in the PFC were significantly increased in both susceptible (*P* < 0.01) and resilient (*P* < 0.01) mice compared with controls (*F*_2,47_ = 8.215, *P* = 0.0009; Figure 2(b)). The autophagy factor ATG12-ATG5 conjugate facilitates the lipidation of members of the LC3 family [34]. The transcripts of *Atg12* in the PFC were significantly increased in both susceptible (*P* < 0.01) and resilient (*P* < 0.001) mice compared with controls, and they were induced to a higher level in resilient mice than in susceptible mice (*P* < 0.05) (*F*_2,47_ = 9.583, *P* = 0.0003; Figure 2(c)). However, the transcript levels of *Atg5* in the PFC were significantly increased only in resilient mice compared with nonstressed controls (*P* < 0.01) (*F*_2,47_ = 9.583, *P* = 0.0003; Figure 2(d)). Moreover, the major autophagosomal marker Lc3b (Map1lc3b) was significantly increased (*P* < 0.01) in resilient mice but unaltered in susceptible mice (*P* = 0.065) compared with nonstressed controls (*F*_2,47_ = 6.953, *P* = 0.0023; Figure 2(e)). The transcripts of p62, a surrogate marker for autophagic degradation, tended to be higher in susceptible mice than in nonstressed controls, but the difference was not statistically significant (*P* = 0.072) (*F*_2,47_ = 4.483, *P* = 0.020; Figure 2(f)). However, the p62 levels were significantly lower in resilient mice than in susceptible mice (*P* < 0.05; Figure 2(f)), suggesting enhanced autophagic flux in the PFC of resilient mice. The FK506-binding protein 51 (FKBP5/FKBP51), a protein known to regulate glucocorticoid receptors, synergizes with antidepressants by enhancing autophagy independent of mTOR [35]. The levels of Fkbp5 mRNA have also been reported to be increased in the amygdala, paraventricular nucleus, and hippocampus under chronic stress [36]. Thus, we examined Fkbp5 transcripts in the mouse PFC after RSD exposure. Fkbp5 transcripts in the PFC were significantly increased in resilient mice (*P* < 0.05) but not in susceptible mice (*P* = 0.054) (*F*_2,42_ = 4.261, *P* = 0.021; Figure 2(g)). The transcript of prefrontal cortical mTOR, a pivotal regulator of autophagy, was undetectable in our real-time PCR assays.

### 3.3. Alteration of Autophagy-Related Genes in the Prefrontal Cortex of Major Depressive Patients

**Figure 3:**
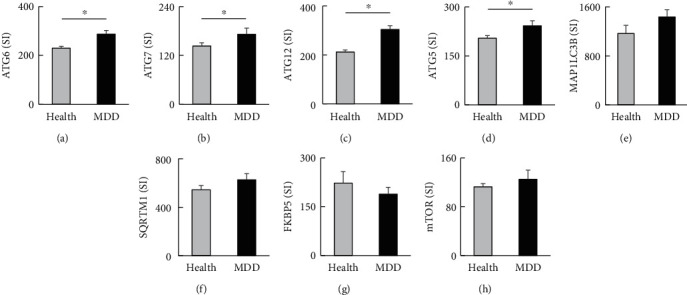
Autophagic signaling changes in the postmortem prefrontal cortex of patients with depression. (a) The expression of ATG6 mRNA in the postmortem tissue. (b) The expression of ATG7 mRNA in the postmortem tissue. (c) The expression of ATG12 mRNA in the postmortem tissue. (d) The expression of ATG5 mRNA in the postmortem tissue. (e) The expression of MAP1LC3B mRNA in the postmortem tissue. (f) The expression of SQRTM1 mRNA in the postmortem tissue. (g) The expression of FKBP5 mRNA in the postmortem tissue. (h) The expression of mTOR mRNA in the postmortem tissue. SI: signal intensity; Health: healthy control; MDD: major depressive disorder. Health, *n* = 56; MDD, *n* = 29. The random variance *t*-test with the Benjamini–Hochberg false discovery (FDR) correction was applied. Data are presented as the mean ± SEM. ^∗^FDR *q* value < 0.05.

Human brain microarray data from the NCBI GEO database were evaluated to determine whether the altered gene expression observed in RSD-exposed mice similarly occurred in postmortem human PFC tissues. Among the autophagy-related genes investigated in the mouse depression model above, patients with MDD (*n* = 29) showed significantly increased transcript levels of *ATG6* (*P* = 0.0004, FDR *q* value = 0.025; Figure 3(a)), *ATG7* (*P* = 0.004, FDR *q* value = 0.045; Figure 3(b)), *ATG12* (*P* = 0.002, FDR *q* value = 0.034; Figure 3(c)), and *ATG5* (*P* = 0.005, FDR *q* value = 0.0497; Figure 3(d)) compared with healthy subjects (*n* = 56). The transcripts of the autophagosome marker *MAP1LC3B* showed a tendency to increase (*P* = 0.072) but were not significantly different (FDR *q* value = 0.201; Figure 3(e)) compared with those in healthy subjects. Furthermore, the signal intensity of *SQRTM1*/*P62* was increased in MDD patients (*P* = 0.043) without statistical significance (*q* value = 0.149; Figure 3(f)). Our findings suggest autophagosome accumulation in the PFC of patients [37]. In addition, in the PFC of MDD patients, *Fkbp5* transcripts tended to decrease (*P* = 0.060) with a statistically significant FDR (*q* value = 0.18; Figure 3(g)). Although decreased mTOR signal was reported in MDD patients [11], transcripts of mTOR increased in patients with MDD compared with controls in our results (*P* = 0.017) (FDR *q* value = 0.089; Figure 3(h)).

### 3.4. Microglial Autophagy Associated with Stress-Induced Depressive-Like Behavior

**Figure 4:**
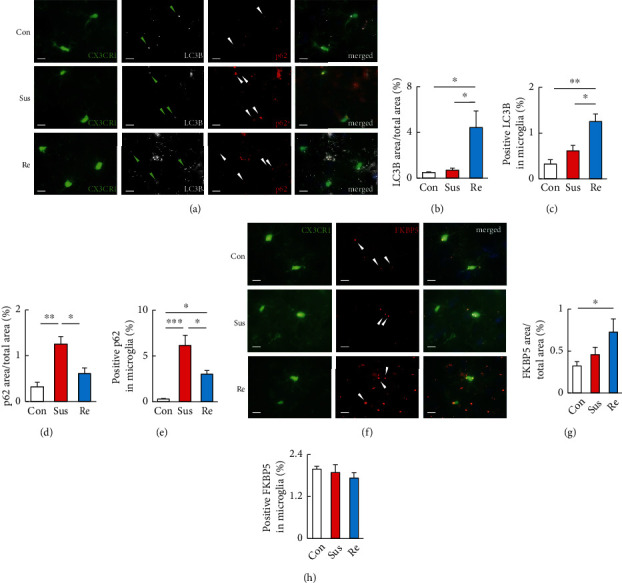
Repeated social defeat stress enhanced autophagy activation in the prefrontal cortex of resilient mice. (a–e) Representative images (a) and quantitative analyses (b–e) of immunostaining for LC3B-puncta colocalized with p62 signals in the PFC of CX3CR1^GFP/+^ mice without RSD (Con) and susceptible and resilient mice 24 h after the last session of RSD. In the merged images in (a), CX3CR1, LC3B, and p62 are shown in green, white, and red, respectively. Scale bars, 10 *μ*m. (b) was determined by calculating the density of the LC3B area/total area. (c) was determined by calculating the density of the positive LC3B signals in each microglia. (e) was determined by calculating the density of the p62 area/total area. (f) was determined by calculating the density of the positive p62 signals in each microglia. (f–h) Representative images (f) and quantitative analyses (g, h) of immunostaining for FKBP5 in the PFC of Cx3CR1^GFP/+^ mice without RSD (Con) and susceptible and resilient mice 24 h after the last session of RSD. In the merged images in (f), CX3CR1 and FKBP5 are shown in green and red, respectively. Scale bars, 10 *μ*m. (g) was determined by calculating the density of the FKBP5 area/total area. (h) was determined by calculating the density of the positive FKBP5 signals in microglia. Con: control mice; Sus: susceptible mice; Re: resilient mice. One-way ANOVA followed by Turkey's post hoc test was applied to all comparisons. Data are presented as the mean ± SEM. Con, *n* = 4; Sus, *n* = 4; Res, *n* = 4. ^∗^*P* < 0.05, ^∗∗^*P* < 0.01, and ^∗∗^*P* < 0.001 vs. Con or Re.

The microglia are the primary glial cells of the innate immune system of the brain, and autophagy in microglia contributes to neurodegenerative diseases [38]. To examine whether autophagy occurs in microglia under chronic stress, microglial GFP (Cx3cr1-GFP) mice were used to confirm the LC3B-positive puncta that colocalized with p62. After one-way ANOVA with Tukey's multiple comparison analyses, LC3B was significantly increased in the PFC of resilient mice compared with nonstressed controls (*P* < 0.05) and susceptible mice (*P* < 0.05), respectively (*F*_2,9_ = 7.107, *P* = 0.014; Figures 4(a) and 4(b)). The increase in LC3B-positive puncta in microglia of resilient mice PFC is significantly higher than that of nonstressed controls (*P* < 0.01) and susceptible mice (*P* < 0.05), respectively (*F*_2,9_ = 13.51, *P* < 0.01; Figures 4(a) and 4(c)). Immunofluorescent signals for p62 were colocalized with LC3B in nonstressed and resilient mice. However, LC3B practically colocalized with overexpressed p62 in susceptible mice (merged in Figure 4(a)). The expression of p62 was significantly more abundant in the PFC of susceptible mice than in the PFC of nonstressed controls (*P* < 0.01) and resilient mice (*P* < 0.05) (*F*_2,9_ = 13.51, *P* = 0.0019; Figures 4(a) and 4(d)). The p62-positive signals in microglia were more abundant in both susceptible (*P* < 0.001) and resilient (*P* < 0.05) mice PFC compared with those in nonstressed controls (*F*_2,15_ = 17.95, *P* < 0.001; Figures 4(a) and 4(e)). However, resilient mice showed significantly less microglial p62 signals than susceptible mice (*P* < 0.05), indicating enhanced autophagic flux in PFC microglia of resilient mice. We also found significantly increased protein levels of FKBP5 in the PFC of resilient mice (*P* < 0.05) but not in the PFC of nonstressed controls (*P* = 0.211) (*F*_2,18_ = 3.653; *P* = 0.047) (Figures 4(g) and 4(h)). In contrast, microglial FKBP5 signals were unaffected in both susceptible and resilient mice compared with controls (*F*_2,18_ = 0.618; *P* = 0.550) (Figures 4(g) and 4(i)).

**Figure 5:**
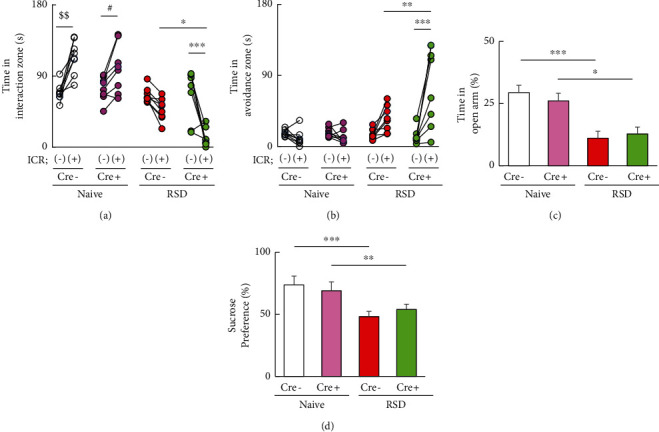
The effect of microglial autophagy deficient on behavior changes. (a, b) The levels of social interaction (a) and social avoidance (b) in Cre-negative mice and Cre^+^ mice with or without RSD. The duration in the interaction (a) or avoidance (b) zone without and with an ICR mouse was analyzed and is shown (*n* = 7). One-way ANOVA followed by Turkey's post hoc test was applied to all comparisons. Data are presented as the mean ± SEM. ^$$^*P* < 0.01 vs. ICR (-) Cre-negative. ^#^*P* < 0.05 vs. ICR (-) Cre^+^. ^∗^*P* < 0.05, ^∗∗∗^*P* < 0.001 vs. ICR (+) Cre-negative. (c) The proportions of the time for the open arms in the elevated plus maze test as an index for anxiety of Cre-negative and Cre^+^ mice (*n* = 7). (d) The proportions of the sucrose intake in the sucrose preference test as an index for depression of Cre-negative and Cre^+^ mice (*n* = 7). One-way ANOVA followed by Turkey's post hoc test was applied to all comparisons. Data are presented as the mean ± SEM. ^∗^*P* < 0.05, ^∗∗∗^*P* < 0.001 vs. Cre-negative or Cre^+^; repeated social defeat, Atg+/+; Cre-negative mice, Atg-/-; and Cre^+^;Atg^flox/flox^ mice. Naive: nonstressed; RSD: repeated social defeat.

We further examined the role of microglial autophagy in stress-induced depressive-like behavior in microglial Atg7-knockout mice (Cre^+^;Atg^flox/flox^). After RSD exposure, stressed Cre-negative mice exhibited resilience (IS scores: 1.54 ± 0.28; *n* = 3) and susceptibility (IS scores: 0.46 ± 0.16; *n* = 4) while all the RSD-stressed Cre+;Atgflox/flox mice showed significantly susceptible behavior (0.51 ± 0.07; *n* = 7). Avoidance behavior was analyzed by one-way ANOVA with Tukey's multiple comparison analyses. The SIT showed that nonstressed controls with intact Atg7 expression (Cre^−^; white circle; *P* < 0.01) and nonstressed microglial Atg7-knockout mice (Cre^+^; pink circle; *P* < 0.05) exhibited increased time in the interaction zone when exposed to CD1 mice (*F*_7,56_ = 19.55, *P* < 0.0001; Figure 5(a)). The stressed microglial Atg7-knockout mice (Cre^+^; green circle) showed significantly decreased time in the interaction zone when exposed to CD1 mice compared with stressed control mice (Cre^−^; red circle; *P* < 0.05) (*F*_7,56_ = 19.55, *P* < 0.0001; Figure 5(a)). In addition, stressed microglial Atg7-knockout mice (Cre^+^; green circle) exhibited increased time in the avoidance zone when exposed to CD1 mice compared with stressed control mice (Cre^−^; red circle; *P* < 0.01) (*F*_7,56_ = 10.46, *P* < 0.0001; Figure 5(b)). These findings demonstrated the role of microglial autophagy in SA. In contrast, in EPM and SPT, both control (Cre^−^) and microglial Atg7-knockout (Cre^+^) mice showed similar decreases in the time spent in the open arm (*P* < 0.001 and *P* < 0.05, respectively; *F*_3,24_ = 10.11, *P* = 0.0002; Figure 5(c)) and a similar decrease in sucrose intake (*P* < 0.001 and *P* < 0.01, respectively; *F*_3,24_ = 11.22, *P* < 0.0001; Figure 5(d)) compared with nonstressed controls of the respective genotypes. Thus, microglial Atg7 deficiency did not affect EPT (Figure 5(d)) or SPT (Figure 5(d)) after RSD exposure. These results demonstrated the selective role of microglial autophagy in chronic stress-induced social avoidance.

## 4. Discussion

Our results determined the increased transcription of the initial autophagy signaling proteins Atgs in the PFC of stressed mice that received RSD. RSD significantly increased the transcript levels of Atgs (*Atg6*, *Atg7*, and *Atg12*) in PFC of both susceptible and resilient mice, which are partially consistent with a previous report that chronic mild unpredictable stress activates hippocampal autophagy in mice [12]. However, enhanced autophagosome formation and autophagosome-lysosome degradation were only observed in resilient mice, whereas susceptible mice (*P* = 0.065) and MDD patients (*P* = 0.072) exhibited an increased tendency of failure of autophagosome formation and its accumulation. The ability to cope with stressful events varies across individuals, with resilient ones being able to control stress and susceptible ones being not. The mechanisms underlying these different stress responses have not yet been clarified. A previous study showed that autophagy plays an essential role in synaptic plasticity injury and cognitive decline [39, 40]. Stressful event-selective reduction of dendritic spine density in the PFC of susceptible mice [41] could partly explain the different feedback of autophagy activation in resilient but not susceptible mice and patients with MDD. Furthermore, the PFC and the ventral tegmental area (VTA) are key brain regions within the neural circuit of the stress response [42]. RSD-induced mTOR phosphorylation significantly increased in the VTA only in the susceptible mice [43]. Similar to our results, FKBP5 was increased only in resilient mice, suggesting that different autophagy regulators may cause an inefficient and enhanced autophagic flux in susceptible and resilient mice, respectively. However, it is still necessary to define whether variations previously observed after RSD in other Atg expressions are differentially modulated in resilient versus susceptible mice to clarify its association with depressive-like behavior.

Autophagy is a degradative pathway that is essential for tissue homeostasis. Previous studies have shown that autophagy is increased not only by promoting autophagosome formation but also by blocking the disruption of autophagic flux [44]. In the CNS, autophagosome accumulation has been reported in a variety of neurodegenerative disorders, such as Alzheimer's disease, Huntington's disease, and Parkinson's disease [45, 46]. After traumatic brain injury, impaired autophagic flux and pathological accumulation of autophagosomes cause neuronal cell death and exacerbate the severity of trauma [47]. However, alterations in autophagic flux in patients with MDD have not been established. Several studies have provided evidence of autophagic molecular changes in patients with MDD. For instance, the peripheral blood mononuclear cells of patients with MDD showed increased expression of autophagy-related genes, such as *BECLIN1* (*ATG6*), *ATG12*, and *LC3* [10]. mTOR signaling was attenuated in the postmortem brains of patients with MDD [11]. These results corroborate with our finding that initial autophagy signaling is activated in patients with MDD. Thus, initial autophagy signaling (*Atg5*, *Atg6*, *Atg7*, and *Atg12*) was elevated but limited without enhanced autophagic flux, such as increased LC3 and P62, in postmortem brains of MDD patients, similar to the RSD-induced susceptible mice. Together, these findings suggest that the induction of initial autophagy followed by impaired autophagic flux results in the pathological accumulation of autophagosomes and ultimately leads to depression. Interestingly, susceptible mouse PFC showed a significantly reduced autophagosome formation but significantly increased accumulation compared with that of resilient mice. Under RSD exposure, the diverse phases in autophagy flux, from the rate of autophagosome formation to the fusion of autophagosome-lysosome and its degradation, need to be elucidated.

Animal and in vitro studies have shown the potential roles of autophagy in the mechanism of antidepressant action. Microglial autophagy deficiency inhibits the behavioral effects of fluoxetine treatment on chronic unpredictable stress [24]. Desipramine elevated the autophagic protein levels of Beclin1 and LC3 in C6 glioma cells [48]. Imipramine stimulated autophagy progression in human U-87MG glioma cells [49], while ketamine promotes neural differentiation of mouse embryonic stem cells via mTOR activation [50, 51]. Thus, antidepressants have diverse effects on autophagy modulation. It is well known that mTOR activation is a crucial regulator of autophagy induction in the nervous system [52]. A previous study showed decreased mTOR protein levels in the postmortem PFC of patients with MDD [11]. However, our analyses of postmortem PFC in MDD patients 5showed different alterations, and mTOR transcripts were undetectable in the PFC of stressed and control mice. Recently, FK506 binding protein 51 (FKBP5) has been linked with autophagy regulators independent of mTOR signaling. Notably, FKBP5 can enhance autophagy and synergize with antidepressant action [53]. Under restraint stress, Fkbp5 mRNA levels were increased in the hippocampus, amygdala, and paraventricular nucleus of the hypothalamus [36]. In our study, the PFC showed significantly increased FKPB5, indicating that variable expression of FKBP5 in a specific brain region may affect different stress response patterns, and it is necessary to focus on the role of FKPB5 in astrocytes or neurons in the future. Moreover, the antidepressant ketamine inhibits mTOR signaling, although its anesthetic and hallucinogenic effects limit its clinical use in most countries. The prefrontal cortical FKBP5 induction may be used as an mTOR-independent antidepressant to prevent depression.

The microglial activation function in pruning and removing dead cells and releasing humoral factors for immune responses may be involved in the pathogenesis of MDD [15, 54, 55]. Recently, various studies have suggested the essential role of autophagy in microglia in the pathophysiology in the CNS. For instance, microglial Agt5 knockdown was sufficient to trigger M1 microglial polarization, while upregulation of autophagy promoted microglial polarization toward the M2 phenotype [22]. Microglial Atg7 deficiency was associated with reduced microglia-mediated neurotoxicity resulting in impaired microglial proinflammatory response [56]. In the animal studies, microglial autophagy is important for refining synapses during development, and defects cause autism spectrum disorder-like behavior [23]. Previous studies have shown that microglial autophagy dysfunction does not exhibit anxiety and depressive-like behavior [24], which is consistent with the lack of effects on EPM and SPT in our results (Figure 5). Furthermore, deficient autophagy in microglia impaired synaptic pruning [23] which may potentially explain the increased susceptibility and aggravated social avoidance in the microglial autophagy deficiency mice by RSD in our results. Additionally, in the CNS, activation of Toll-like receptors (TLRs) in microglia leads to impaired microglial autophagy [57]. Microglial TLR2/4 deficiency also abolishes RSD-induced social avoidance [21], suggesting that microglial autophagic regulation via TLR activation may affect stress-induced avoidance changes. In addition, enhanced autophagy in the PFC may occur in cells other than microglia in resilient mice. Thus, the behavioral roles of autophagy in astrocytes and neurons in anxiety-depressive-like behaviors remain to be studied [58].

## 5. Conclusion

Repeated social stress induced the initial activation of autophagy in the PFC of stressed mice and patients with MDD. The enhanced autophagic flux was only determined in the prefrontal cortical microglia of resilient mice, revealing the relationship between autophagy and stress-induced depressive behavior. Furthermore, microglia autophagy deficiency impaired stress-induced avoidance behavior, but not anxiety and depressive-like behaviors. These findings help to better understand microglial autophagic functions for stress and depression and might lead to the autophagy-based development of antidepressants.

## Data Availability

The data that support the findings of this study are available on request from the corresponding author.

## Additional Points

*Main Points*. (i) Repeated social stress (RSD) induced initial autophagy signals and enhanced autophagic flux in the stress resilience. (ii) Patients with depression exhibited enhanced initial autophagy signaling. (iii) Autophagy deficiency in microglia aggravates RSD-induced avoidance.
